# Effect of Intravitreal Anti-Vascular Endothelial Growth Factor Therapy on the Risk of Arterial Thromboembolic Events: A Meta-Analysis

**DOI:** 10.1371/journal.pone.0041325

**Published:** 2012-07-19

**Authors:** Jin-Wei Cheng, Shi-Wei Cheng, Guo-Cai Lu, Rui-Li Wei

**Affiliations:** 1 Department of Ophthalmology, Shanghai Changzheng Hospital, Second Military Medical University, Shanghai, China; 2 School of Life Sciences, Ludong University, Yantai, China; 3 Center for New Drug Evaluation, Institute of Basic Medical Science, Second Military Medical University, Shanghai, China; Univeristy of Melbourne, Australia

## Abstract

**Background:**

Intravitreal anti-vascular endothelial growth factor (VEGF) monoclonal antibodies are used in ocular neovascular diseases. A consensus has emerged that intravenous anti-VEGF can increase the risk of arterial thromboembolic events. However, the role of intravitreal anti-VEGF in arterial thromboembolism is controversial. Therefore, we did a systematic review and meta-analysis to investigate the effects of intravitreal anti-VEGF on the risk of arterial thromboembolic events.

**Methods:**

Electronic databases were searched to identify relevant randomized clinical trials comparing intravitreal anti-VEGF with controls. Criteria for inclusion in our meta-analysis included a study duration of no less than 12 months, the use of a randomized control group not receiving any intravitreal active agent, and the availability of outcome data for arterial thromboembolic events, myocardial infarction, cerebrovascular accidents, and vascular death. The risk ratios and 95% CIs were calculated using a fixed-effects or random-effects model, depending on the heterogeneity of the included studies.

**Results:**

A total of 4942 patients with a variety of ocular neovascular diseases from 13 randomized controlled trials were identified and included for analysis. There was no significant difference between intravitreal anti-VEGF and control in the risk of all events, with risk ratios of 0.87 (95% CI, 0.64 to 1.19) for arterial thromboembolic events, 0.96 (95% CI, 0.55–1.68) for cerebrovascular accidents, 0.69 (95% CI 0.40–1.21) for myocardial infarctions, and 0.68 (95% CI, 0.37–1.27) for vascular death.

**Conclusions:**

The strength evidence suggests that the intravitreal use of anti-VEGF antibodies is not associated with an increased risk of arterial thromboembolic events.

## Introduction

Angiogenesis, a process involving the proliferation of new blood vessels, plays a crucial role in many pathologic states [1]. This process is mainly driven by vascular endothelial growth factor (VEGF), whose signaling pathway has been a target of many new antiangiogenic agents [2]. Currently used monoclonal antibodies against VEGF included pegaptanib, ranibizumab, and bevacizumab. Bevacizumab (Avastin®) is a recombinant full length humanized antibody that binds to all types of VEGF and is used successfully in the treatment of many types of malignancy as a systemic drug [3], [4]. Pegaptanib (Macugen®), a 28-base ribonucleic acid aptamer covalently linked to two branched 20-kD polyethylene glycol moieties, binds to extracellular VEGF, specifically the 165-amino-acid isoform (VEGF-165), and antagonizes its biological effects [5]. Ranibizumab (Lucentis®) is a recombinant humanized monoclonal antibody Fab that neutralizes all active forms of VEGF-A [6]. All three anti-VEGF agents have been proven promise in the treatment of various ocular neovascular diseases, such as age-related macular degeneration, diabetic retinopathy, and retinal vein occlusion [7], [8].

Because VEGF plays many roles in physiologic processes, its inhibition could have potentially serious systemic consequences. While the use of intravenous bevacizumab is recognized to be associated with an increased risk of arterial and venous thromboembolic events [9], [10], it is controversial whether intravitreal anti-VEGF agents contribute to the development of arterial thromboembolic events, such as myocardial infarction and cerebrovascular accidents, common comorbidities leading to mortality in patients with ocular neovascular diseases [11], [12]. A pooled analysis from three randomized clinical trials (RCTs) that included 859 patients with age-related macular degeneration showed that intravitreal ranibizumab was associated with an increased risk of cerebrovascular accidents (odds ratios [OR], 3.24; 95% confidence interval [CI], 0.96–10.95; P = 0.045), when compared with sham treatment, whereas there was no apparent association between intravitreal ranibizumab and myocardial infarction (OR, 0.61 [95%CI, 0.29–1.29]; P = 0.193) [13]. Because the number of patients included in this analysis is limited, the contribution of intravitreal anti-VEGF therapy to arterial thromboembolic events remains poorly defined.

**Figure 1:**
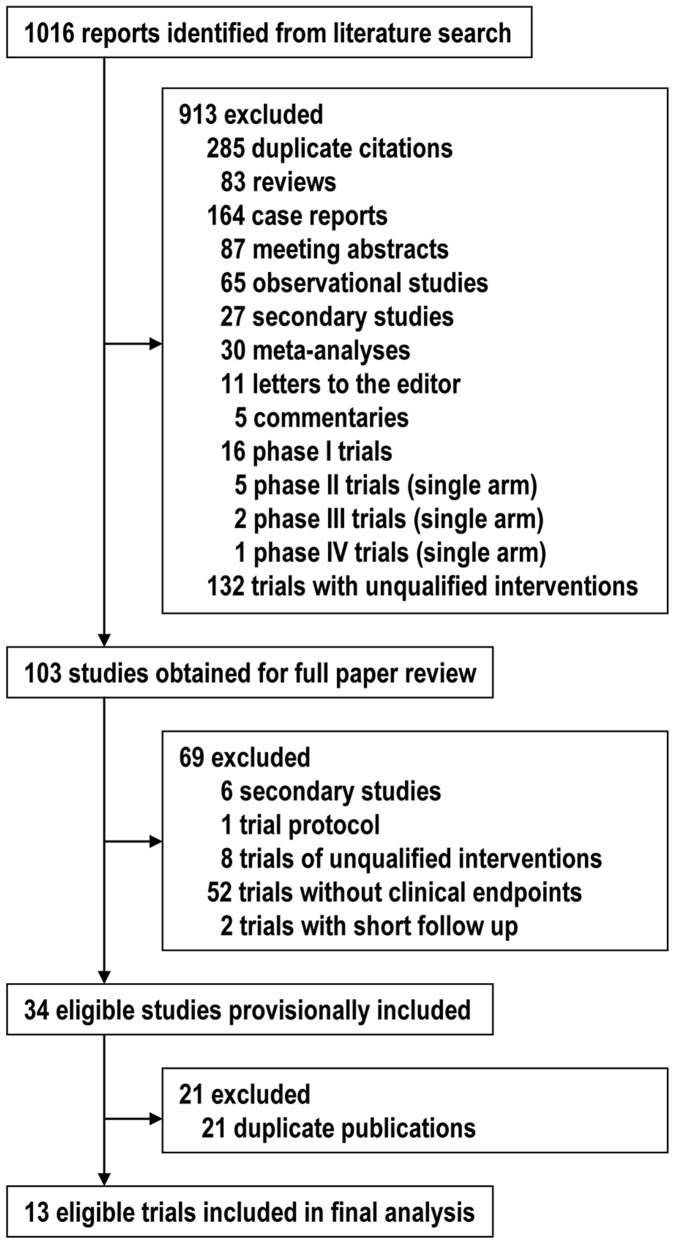
Flow diagram showing citations retrieved from literature searches and number of trials included in the meta-analysis.

**Table 1:** Characteristics of randomized controlled clinical trials in the meta-analysis.

Acronym	Design	Treatment arm	Control arm	Concurrent treatment	Number of patients	Disease	Mean age (range, years)	Women (%)	Follow up (months)	Lost tofollow-up (%)	Quality score
VISION^22^	DB-P	Pegaptanib	Placebo	Verteporfin	1190	Neovascular AMD	NA	696 (58.5)	12	11.5	5
MARINA^23^	DB-P	Ranibizumab	Placebo	Verteporfin	716	Neovascular AMD	77 (52–95)	464 (64.8)	24	14.1	5
FOCUS^24^	SB-P	Ranibizumab	Placebo	Verteporfin	162	Neovascular AMD	74 (50–93)	86 (53.1)	24	14.8	4
ANCHOR^25^	DB-P	Ranibizumab	Verteporfin	Laser	423	Neovascular AMD	77 (53–97)	211 (49.9)	24	22.2	5
BOLT^26^	SB-P	Bevacizumab	Laser	None	80	DME	64 (40–86)	25 (31.3)	12	2.5	4
ABC Trial^27^	DB-P	Bevacizumab	Placebo, verteporfin, pegaptanib	None	131	Neovascular AMD	81 (NA)	80 (61.1)	12	4.8	5
PIER^28^	DB-P	Ranibizumab	Placebo	Verteporfin	184	Neovascular AMD	78 (54–94)	110 (59.8)	24	16.8	5
RESOLVE^29^	DB-P	Ranibizumab	Placebo	None	151	DME	64 (32–85)	70 (46.4)	12	12.6	5
BRAVO^30^	DB-P	Ranibizumab	Placebo	Laser	397	BRVO	66 (26–91)	185 (46.6)	12	10.3	5
CRUISE^31^	DB-P	Ranibizumab	Placebo	None	392	CRVO	68 (20–91)	169 (43.1)	12	11.0	5
DRCR^32^	DB-P	Ranibizumab	Placebo, triamcinolone	Laser	691	DME	63 (NA)	304 (44.0)	24	6.4	5
RESTORE^33^	DB-P	Ranibizumab	Placebo	Laser	345	DME	63 (NA)	144 (41.7)	12	12.2	5
Macugen 1013^34^	DB-P	Pegaptanib	Placebo	Laser	260	DME	62 (20–83)	111 (42.7)	24	32.5	5

NA = data not available. DB-P  =  double blind parallel; SB-P  =  single blind parallel. AMD  =  age-related macular degeneration; DME  =  diabetic macular edema; CRVO  =  central retinal vein occlusion; BRVO  =  branch retinal vein occlusion.

Recently, many more RCTs of intravitreal anti-VEGF therapy in ocular neovascular diseases have been performed. However, no significant association between intravitreal anti-VEGF therapy and arterial thromboembolic events has been shown in any RCTs. We hypothesized that these studies were not powered sufficiently to reveal a significantly increased risk due to the low incidences of arterial thromboembolic events. Therefore, we performed a systematic review of the published RCTs for a meta-analysis to determine the risk of arterial thromboembolic events associated with intravitreal anti-VEGF treatment.

**Figure 2:**
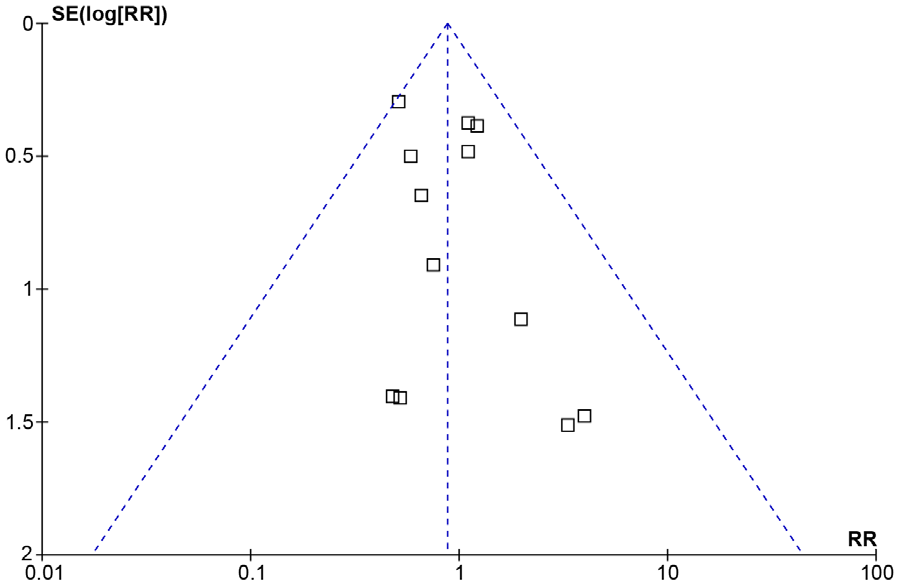
Funnel plot of studies on arterial thromboembolic events.

## Methods

### Data Source

Published RCTs were identified through a comprehensive search of PubMed, EMBASE, and the Cochrane central register of controlled trials, each from inception to October 31, 2011. The search combined terms related to drugs (bevacizumab, pegaptanib, and ranibizumab), and terms related to diseases (macular degeneration, diabetic retinopathy, macular edema, retinal vein occlusion, retinal neovascularization, and choroidal neovascularization), with a filter to restrict results to clinical trial. The reference lists of identified articles were examined for additional publications.

**Figure 3:**
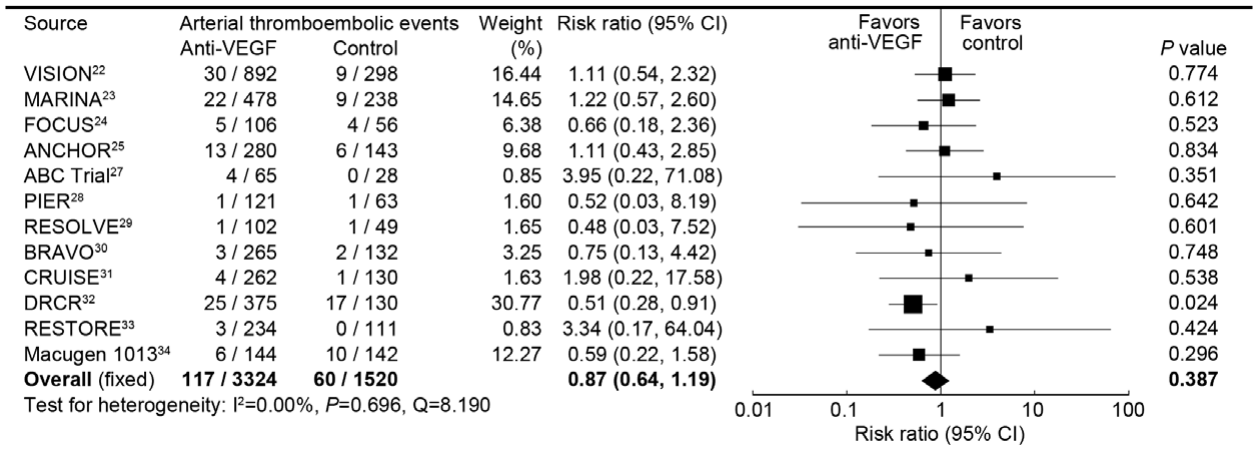
Risk ratio of arterial thromboembolic events associated with intravitreal anti-VEGF treatment compared with control treatment.

**Table 2:** Risk ratio of arterial thromboembolic events.

		Number of events/total number (%)			
	No of Trials	Anti-VEGF	Control	Risk ratio (95% CI)	P value
**Arterial thromboembolic events**					
Overall	12	117/3324 (3.5)	60/1520 (3.9)	0.87 (0.64, 1.19)	0.387
Neovascular AMD	6	75/1942 (3.9)	29/826 (3.5)	1.14 (0.73, 1.70)	0.615
DME	4	35/885 (4.1)	28/432 (6.5)	0.58 (0.36, 0.94)	0.028
RVO	2	7/527 (1.3)	3/262 (1.2)	1.16 (0.30, 4.45)	0.829
Ranibizumab	9	77/2223 (3.5)	41/1052 (3.9)	0.83 (0.58, 1.19)	0.315
Pegaptanib	2	36/1036 (3.5)	19/440 (4.3)	0.89 (0.50, 1.59)	0.696
Bevacizumab	1	4/65 (6.2)	0/28 (0.0)	3.96 (0.22, 71.08)	0.351
**Cerebrovascular accidents**					
Overall	10	32/2288 (1.4)	15/1169 (1.3)	0.96 (0.55, 1.68)	0.891
Neovascular AMD	3	18/864 (2.1)	4/437 (0.9)	2.10 (0.76, 5.83)	0.155
DME	5	11/897 (1.2)	10/470 (2.1)	0.51 (0.24, 1.09)	0.083
RVO	2	3/527 (0.6)	1/262 (0.4)	1.16 (0.17, 7.82)	0.877
Ranibizumab	8	30/2102 (1.2)	17/1052 (1.6)	0.96 (0.53, 1.74)	0.899
Pegaptanib	1	2/144 (1.4)	1/142 (0.7)	1.97 (0.18, 21.51)	0.577
Bevacizumab	1	0/42 (0.0)	1/38 (2.6)	0.30 (0.01, 7.21)	0.460
**Myocardial infarction**					
Overall	11	29/2432 (1.2)	20/1222 (1.6)	0.69 (0.40, 1.21)	0.195
Neovascular AMD	5	18/1050 (1.7)	10/528 (1.9)	0.86 (0.41, 1.81)	0.700
DME	4	8/855 (0.9)	8/432 (1.9)	0.46 (0.18, 1.23)	0.121
RVO	2	3/527 (0.6)	2/262 (0.8)	0.75 (0.13, 4.43)	0.747
Ranibizumab	9	27/2223 (1.2)	17/1052 (1.6)	0.73 (0.41, 1.31)	0.292
Pegaptanib	1	0/144 (0.0)	3/142 (2.1)	0.14 (0.01, 2.70)	0.194
Bevacizumab	1	2/65 (3.1)	0/28 (0.0)	2.20 (0.11, 44.34)	0.608
**Vascular death**					
Overall	4	25/1198 (2.1)	15/539 (2.8)	0.68 (0.37, 1.27)	0.225
Neovascular AMD	3	12/823 (1.4)	7/409 (1.7)	0.79 (0.31, 2.01)	0.626
DME	1	13/375 (3.5)	8/130 (6.2)	0.55 (0.22, 1.35)	0.192
Ranibizumab	3	23/1133 (2.0)	15/511 (2.9)	0.63 (0.33, 1.20)	0.159
Bevacizumab	1	2/65 (3.1)	0/28 (0.0)	2.20 (0.11, 44.34)	0.608

AMD  =  age-related macular degeneration; DME  =  diabetic macular edema; RVO  =  retinal vein occlusion.

We reviewed each publication and only the most recent or complete report of clinical trials was included when duplicate publications were identified. Efforts also were made to contact the investigators when relevant data were not clear.

**Figure 4:**
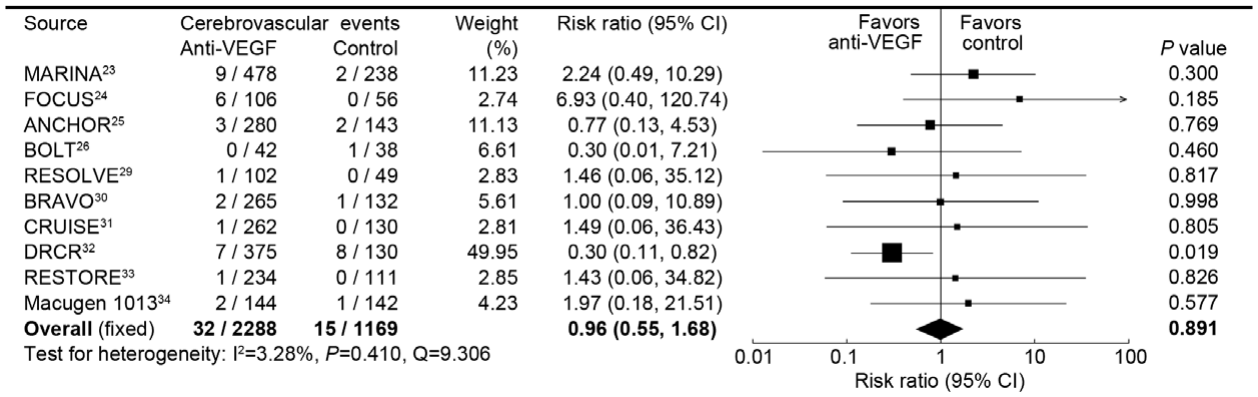
Risk ratio of cerebrovascular accidents associated with intravitreal anti-VEGF treatment compared with control treatment.

### Study Selection

The goal of this study was to determine whether intravitreal anti-VEGF therapy contributes to the development of arterial thromboembolic events. Therefore, only RCTs with a direct comparison between patients treated with and without intravitreal injection of anti-VEGF agents were included for analysis. Specifically, clinical trials fulfilling the following criteria were included in the meta-analysis: (i) study design – randomized clinical trials, which all should have adequate IRB review and consent processes; (ii) population – patients with ocular neovascular diseases, such as age-related macular degeneration, diabetic retinopathy, and retinal vein occlusion; (iii) intervention – intravitreal anti-VEGF agents versus control, and the use of a randomized control group not receiving any intravitreal active agent; (iv) outcome measurement – the incidence of arterial thromboembolic events, myocardial infarction, cerebrovascular accidents, and vascular death; (v) duration – the minimum length of follow up was 12 months.

**Figure 5:**
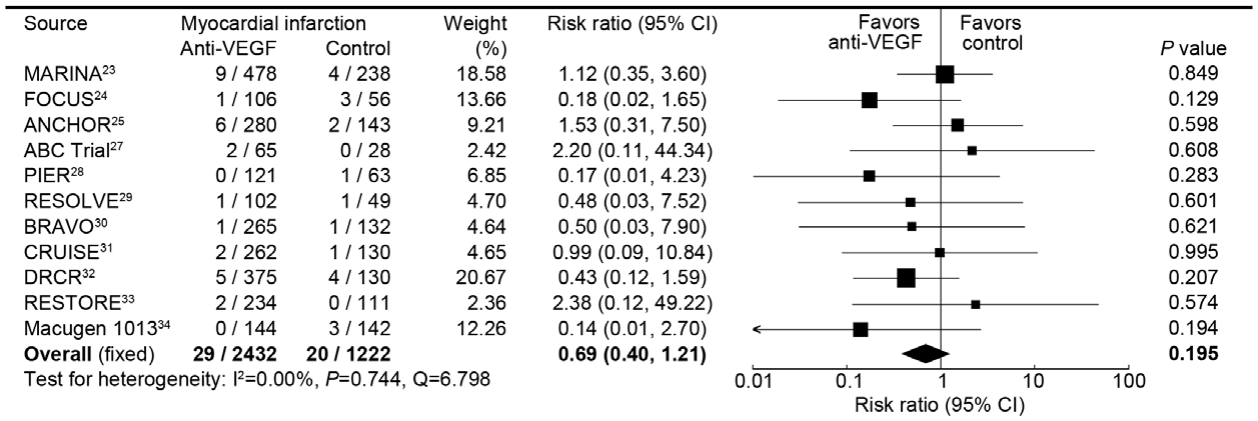
Risk ratio of myocardial infarctions associated with intravitreal anti-VEGF treatment compared with control treatment.

After completion of the searches, two review authors (JWC and SWC) working independently assessed the titles and abstracts of all obtained reports for a rough judgment of an article’s eligibility. The full text copies of possibly and definitely relevant trials were obtained and assessed by the three authors independently according to the definitions in the criteria. Only trials meeting these criteria were assessed for methodological quality.

**Figure 6:**
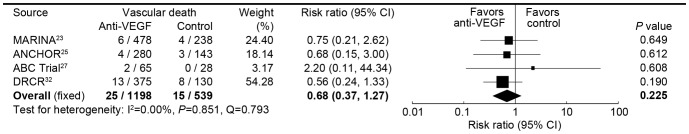
Risk ratio of vascular death associated with intravitreal anti-VEGF treatment compared with control treatment.

### Data Extraction and Clinical Endpoints

Data extraction was performed by two reviewers (JWC and SWC) independently. Any disagreement was resolved by discussion. For each study and each type of treatment, the following data were extracted: information on study design (whether randomization, allocation concealment, intention to treat analysis, double blind or single blind, parallel or crossover), location of trial, length of study, sample size, patient age, sex, race, type of diagnosis, and events of arterial thromboembolic events, myocardial infarction, cerebrovascular accidents, and vascular death.

The clinical endpoints included arterial thromboembolic events, non-fatal cerebrovascular accidents, non-fatal myocardial infarction, and vascular death. Arterial thromboembolic events included nonfatal myocardial infarction, nonfatal stroke, and death from a vascular or unknown cause, on the basis of the classification system of the Antiplatelet Trialists’ Collaboration (APTC) [14]. All reported strokes, transient ischemic attacks, or cerebral ischemic incidents were regarded as cerebrovascular accidents.

### Qualitative Assessment

Two authors (in duplicate by JWC and SWC) used standard criteria (allocation concealment, blinding, intention to treat analysis, loss to follow-up) to appraise study quality, in addition to quantitative quality assessment by using the scoring system developed by Jadad [15]. The quality scoring system was followed as: (i) allocation concealment, coded as adequate (1 score), inadequate or unclear (0 score); (ii) blinding, coded as double-blind (2 scores), single-blind (1 score), and open label (0 score); (iii) intention to treat analysis, coded as used (1 score), not used or unable to assess (0 score); and (iv) lost to follow-up, coded as given (1 score), and not given (0 score). “Poor quality” refers to a Jadad score less than 3, and the impact of excluding low quality studies was assessed by a sensitivity analysis.

### Statistical Analysis

Outcome measure was assessed on an intent-to-treat (ITT) basis, the ITT population comprising all randomized patients who received a minimum of one dose of active treatment and provided a valid baseline measurement.

All statistical analyses were performed using version 2 of the Comprehensive Meta-analysis program (Biostat, Englewood Cliffs, New Jersey). For each study, risk ratio (RR) of arterial thromboembolic events, non-fatal cerebrovascular accidents, non-fatal myocardial infarction, and vascular death with exact 95% CI were calculated. The heterogeneity across all eligible comparisons was estimated using the *Χ* ^2^-based Q statistic. Heterogeneity was checked by *P*-value [16]. *I* ^2^ metrics, which quantify heterogeneity irrespective of the number of studies, were also reported [17]. If no heterogeneity detected (*P*>0.1), we combined the results in a meta-analysis using the Mantel-Haenszel fixed effects model [18], otherwise, the DerSimonian-Laird random effects model were used to pool the data after exploring the causes of heterogeneity [19], [20].

We constructed standard funnel plots to investigate the potential for publication bias, by examining visually the asymmetry. Furthermore, Egger’s linear regression method was used to detect the presence of publication bias regarding primary endpoint (arterial thromboembolic events) [21].

## Results

The flow of the randomized controlled trials included in our analysis is shown in the **Figure 1**. We reviewed the full text of 103 articles from 1016 studies identified from our initial literature search. After excluding secondary studies, trial protocols, trials of unqualified interventions, trials without clinical endpoints, and duplicate publications, totally 13 randomized controlled trials were included in the final meta-analysis (**Table 1**) [22]–[34].

All trials had a prospective, parallel design. Randomized treatment allocation sequences were generated in all trials. Eleven trials were double-blinded, and two other trials were single-blinded. Nine trials had placebo as controls; two other trials had active controls; the rest of the trials were placebo and active controls. Patients were analyzed by the intention to treat principle in all trials. The quality of all the trials was acceptable: eleven trials scored 5, two trials scored 4.

Funnel plot for the studies on arterial thromboembolic events was qualitatively symmetrical (**Figure 2**), and no publication bias was detected for the primary endpoint by Egger’s test (one-tailed, P = 0.13; two-tailed, P = 0.27).

There were 38 patients treated with pegaptanib in ABC Trial [27], and 142 patients treated with triamcinolone in DRCR study [32]; we excluded the two arms from the final analysis. Therefore, a total of 4942 patients from 13 randomized clinical trials were included for analysis. The baseline characteristics of patients in the 13 studies are summarized in **Table 1**. Six studies included neovascular age-related macular degeneration, five included diabetic macular edema, and each one included central retinal vein occlusion and branch retinal vein occlusion.

Our meta-analysis calculated the overall risk ratio for arterial thromboembolic events associated with intravitreal anti-VEGF treatment compared with control treatment, and twelve trials were included in this analysis (**Figure 3**). There were 117 (3.5%) arterial thromboembolic events of 3324 patients in the intravitreal anti-VEGF group, and 60 (3.9%) of 1520 patients in the control group. No significant heterogeneity was found in this analysis. Intravitreal anti-VEGF therapy was not associated with the risk of arterial thromboembolic events, with a pooled risk ratio of 0.87 (95% CI, 0.64 to 1.19) using a fixed-effects model. Analysis using the random effects model similarly showed no association between intravitreal anti-VEGF therapy and the risk of arterial thromboembolic events (pooled risk ratio 0.83, 0.61 to 1.13). **Table 2** lists the risk ratios and 95% confidence intervals for arterial thromboembolic events from all the trials; results from the pooled group according to the type of diseases and the type of interventions are shown separately.

The results of the meta-analysis for cerebrovascular accidents are shown in **Figure 4**, and ten trials were included in this analysis, involving a total of 3457 patients. 32 (1.4%) of 2288 patients in receiving intravitreal anti-VEGF experienced cerebrovascular accidents, compared with 15 (1.3%) of 1169 patients receiving control. There was not a significant heterogeneity in this analysis. Intravitreal anti-VEGF was not associated with the risk of cerebrovascular accidents, with a pooled RR of 0.96 (0.55 to 1.68) by fixed effects analysis and 0.83 (0.44 to 1.57) by random effects analysis. **Table 2** shows the sub-pooled risk ratio, which also suggested that intravitreal anti-VEGF was not associated with the risk of cerebrovascular accidents.

Eleven trials comparing intravitreal anti-VEGF with control reported the rate of myocardial infarction, with a total of 3654 patients included in this analysis. Myocardial infarctions occurred in 29 (1.2%) of 2432 patients receiving intravitreal anti-VEGF, and 20 (1.6%) of 1222 patients receiving control. There was no significant difference between anti-VEGF and control in the risk of myocardial infarctions, with a risk ratio being 0.69 (0.40–1.21) by fixed-effects analysis and 0.70 (0.39 to 1.28) by random effects analysis (**Figure 5**). Intravitreal anti-VEGF was also not associated with the risk of myocardial infarctions for neovascular age-related macular degeneration, diabetic macular edema, and retinal vein occlusion (**Table 2**).

**Figure 6** shows the results of the meta-analysis for vascular death comparing anti-VEGF with control. There were 25 (2.1%) of 1198 patients allocated to treatment with intravitreal anti-VEGF, and 15 (2.8%) of 539 patients allocated to control, who experienced vascular death. No significant heterogeneity was found in this analysis. Intravitreal anti-VEGF was not associated with the risk of vascular death, with a risk ratio of 0.68 (0.37–1.27) from the fixed-effects model, and 0.66 (0.35 to 1.24) from the random-effects model. Sub-group analyses using the fixed-effects model also suggested that intravitreal anti-VEGF was not associated with the risk of vascular death (**Table 2**).

## Discussion

The results of this programme of prospectively designed overviews of data from 13 randomized clinical trials revealed that, as compared with control, intravitreal anti-VEGF therapy was not associated with the risk of arterial thromboembolic events, non-fatal cerebrovascular accidents, non-fatal myocardial infarction, and vascular death.

The previous meta-analyses suggested the use of intravenous bevacizumab was recognized to be associated with an increased risk of arterial and venous thromboembolic events [9], [10]. Because of the high association of the risk of cardiovascular events with age-related macular degeneration, diabetes, and retinal vein occlusion [35]–[37], the results of a previous meta-analysis [13], which revealed intravitreal anti-VEGF was also associated with an increased risk of cerebrovascular accidents, was worrisome. However, another previous systematic review [38], as well as two non-randomized studies [39], [40], suggested that intravitreal anti-VEGF use was not associated with increased risks of mortality, myocardial infarction, or stroke. Therefore, the success to detect such an increase in cerebrovascular accidents risk is likely due to the limited number of trials included for the analysis. Furthermore, risk ratios might be affected by small changes in the classification of events, due to the results based on a relatively small number of events.

In the overview for arterial thromboembolic events, no difference in this risk between the arms receiving intravitreal anti-VEGF and control, with the 95% confidence interval included an up to 19% increased risk of arterial thromboembolic events down to a 36% reduction with intravitreal anti-VEGF. In the overviews for non-fatal cerebrovascular accidents, non-fatal myocardial infarction, and vascular death, there was also no clear difference between intravitreal anti-VEGF and control.

In the present meta-analysis, several ocular neovascular diseases, such as age-related macular degeneration, diabetic retinopathy, and retinal vein occlusion, were included. Sensitivity analysis was undertaken to evaluate the variation of the risk of arterial thromboembolic events with anti-VEGF among different diseases. Intravitreal anti-VEGF significantly decreased the risk of arterial thromboembolic events by 32% in patients with diabetic macular edema, with the 95% confidence intervals of 6% to 64%; no difference in this risk was detected in patients with neovascular age-related macular degeneration and retinal vein occlusion. In patients with diabetes mellitus, increased VEGF-mediated angiogenesis has been implicated in retinopathy and nephropathy, whereas a defective angiogenic response to ischemia, which might be attributable to a VEGF signaling defect in which there is reduced receptor signaling despite higher ligand expression, could lead to poor clinical outcomes [41]. Therefore, the targets within the system that lead to altered VEGF signaling, such as low dose systemic anti-VEGF, may be beneficial in diabetic patients.

The sensitivity analysis according to the type of diseases showed that intravitreal anti-VEGF increased the risks of cerebrovascular accidents by 52% in neovascular age-related macular degeneration, with the 95% confidence intervals of -32% to 83%. However, the point estimates of all three trials were distributed across the 1.0 risk ratio [23]–[25]. Two estimates have shown a possible risk of cerebrovascular accidents of intravitreal anti-VEGF [23], [24]. However, a larger epidemiological study found that no statistically significant relationship between intravitreal anti-VEGF use and stroke [39]. Therefore, the small differences of cerebrovascular accidents between intravitreal anti-VEGF and placebo in the two trials might be due to chance finding, but not drug-related [38].

Although we tried to conduct a thorough review of the existing literature, this present analysis has limitations inherent to any systematic review. First, the incidences of arterial thromboembolic events showed significant heterogeneity among the included studies. This may reflect differences in sample sizes, disease types, interventions, concomitant treatment, study durations, and many other factors among these studies. Despite these differences, the risk ratios reported by all of these studies showed remarkable homogeneity. In addition, combination data by using a random-effects model may be able to achieve more conservative estimates. Second, the included trials were done at various clinical centers, and the ability to detect arterial thromboembolic events and the classification of events might vary among these institutions, which could result in a bias of reported incidence rates. Third, only published studies were included in the present meta-analysis. To avoid the publication bias, we searched in multiple databases. In addition, to find potential publication biases, we explored asymmetry in funnel plots and detect heterogeneity using Egger’s linear regression, and no publication bias was found. Finally, the findings of this meta-analysis are based on the study level, not on patient-level source data, and some confounding factors cannot be properly assessed and incorporated into the results.

Despite these limitations, the strength evidence from the present meta-analysis data suggests that the intravitreal use of anti-VEGF agents is not associated with an increased risk of arterial thromboembolic events.
